# Hypoxia-Induced Fibroblast IL-6 Promotes Immunosuppressive Macrophage Phenotypes in Pancreatic Cancer

**DOI:** 10.3390/cells15080683

**Published:** 2026-04-13

**Authors:** Sean Hannifin, Ashley M. Mello, Tenzin Ngodup, Nam Hoon Kim, Marina Pasca di Magliano, Kyoung Eun Lee

**Affiliations:** 1 Program in Immunology, University of Michigan, Ann Arbor, MI 48109, USA; hannifin@umich.edu (S.H.);; 2 Department of Pharmacology, University of Michigan, Ann Arbor, MI 48109, USA; tenzin@umich.edu (T.N.);; 3 Rogel Cancer Center, University of Michigan, Ann Arbor, MI 48109, USA; 4 Department of Surgery, University of Michigan, Ann Arbor, MI 48109, USA; 5 Department of Cell and Developmental Biology, University of Michigan, Ann Arbor, MI 48109, USA

**Keywords:** hypoxia, pancreatic cancer, macrophage, fibroblast, IL6, ARG1, JAK

## Abstract

Pancreatic ductal adenocarcinoma (PDAC) is a lethal malignancy characterized by a dense fibroinflammatory stroma and profound hypoxia. Using pancreatic stellate cell-tumor organoid coculture models and single-cell RNA sequencing analyses, we uncover that hypoxia-driven fibroblast reprogramming promotes immunosuppressive macrophage phenotypes in PDAC. Mechanistically, hypoxia acts through tumor-fibroblast crosstalk to increase IL-6 expression in fibroblasts; in turn, fibroblast-derived IL-6 induces expression of arginase 1 (ARG1), a key mediator of immunosuppression, in macrophages via activation of the JAK/STAT signaling pathway. Consistent with these findings, macrophages enriched for hypoxia signatures are strongly associated with elevated immunosuppression programs and IL6/JAK/STAT3 signaling signatures in PDAC. Our study reveals a paracrine mechanism by which hypoxia coordinates tumor cell, fibroblast, and macrophage interactions to promote immune suppression in PDAC.

## 1. Introduction

Pancreatic ductal adenocarcinoma (PDAC) is a lethal disease with a five-year survival rate of 13% [1]. A hallmark of PDAC is its extensive fibroinflammatory stroma, which contributes to resistance to chemotherapy and immunotherapy [2,3]. Macrophages and fibroblasts are the predominant cell types within the PDAC stroma, and both exhibit substantial phenotypic and functional plasticity and heterogeneity [2].

Macrophages within tumors, termed tumor-associated macrophages (TAMs), display the ability to suppress T cell recruitment and function as well as regulate other immune populations and tumor cells [4,5,6]. Single-cell RNA sequencing (scRNA-seq) has revealed TAM heterogeneity beyond the traditional M1/M2 classification, shaped by ontogeny, tissue context, and environmental cues [7,8]. TAMs represent a dominant immunosuppressive population in PDAC, and their abundance negatively correlates with patient survival [9].

Cancer-associated fibroblasts (CAFs) comprise distinct subtypes, including myofibroblastic CAFs (myCAFs) and inflammatory CAFs (iCAFs). MyCAFs are primarily involved in extracellular matrix deposition and remodeling, whereas iCAFs secrete high levels of inflammatory cytokines and chemokines [10,11]. We previously demonstrated that hypoxia, a condition of insufficient oxygen (O_2_) availability, promotes the iCAF phenotype through upregulation of tumor-derived IL1α [12].

PDAC is characterized by low vascular density and profound hypoxia [13,14]. Although both tumor and stromal cells experience hypoxia, the impact of hypoxia on stromal cell function and tumor–stroma crosstalk remains incompletely understood. Here, we show that TAMs enriched for hypoxia-responsive gene signatures exhibit elevated immunosuppression programs in PDAC. Using three-dimensional (3D) cocultures of pancreatic stellate cells (PSCs), a precursor population of CAFs, and cancer cells, we demonstrate that hypoxia reprograms PSCs to secrete factors that shift macrophages toward immunosuppressive phenotypes. We identify PSC-derived IL-6 as a critical mediator of this effect through JAK/STAT signaling in macrophages. Together, our findings define a mechanism by which hypoxia promotes immunosuppressive macrophage polarization via fibroblast reprogramming in PDAC.

## 2. Materials and Methods

### 2.1. Mice

All animal protocols were reviewed and approved by the Institutional Animal Care and Use Committee of the University of Michigan (protocol number: PRO00012213). Wild-type (WT) C57BL/6 mice (stock #000664, Jackson Laboratory, Bar Harbor, ME, USA) were used for primary cell isolation at 8–12 weeks of age, including both male and female mice.

### 2.2. Cell Lines and Cell Culture

All cell lines were maintained in DMEM (#MT10017CV, Corning, Glendale, AZ, USA) containing 10% heat-inactivated FBS (#A5209501, Gibco, Waltham, MA, USA) and 1% penicillin–streptomycin (#15140122, Gibco). Cells were routinely tested for mycoplasma using the MycoAlert PLUS Mycoplasma Detection Kit (#LT07-710, Lonza, Basel, Switzerland). The mT3 pancreatic cancer cell line (provided by Dr. David A. Tuveson) [15] was derived from primary mouse KPC (*Kras^LSL-G12D/+^*; *Trp53^LSL-R172H/+^*; *Pdx1-Cre*) PDAC.

### 2.3. PSC Isolation and 3D Culture

PSCs were isolated from WT mice (#000664, Jackson Laboratory, Bar Harbor, ME, USA) as previously described [12]. Briefly, pancreatic tissue was minced and digested for 30 min at 37 °C in Gey’s balanced salt solution (GBSS) containing 0.05% collagenase P (#11213857001, Sigma-Aldrich, St. Louis, MO, USA), 0.02% pronase (#10165921001, Sigma-Aldrich, St. Louis, MO, USA), and 0.1% DNase I (#10104159001, Sigma-Aldrich, St. Louis, MO, USA). The digested tissue was filtered through a 100 μm cell strainer (#352360, Falcon, Glendale, AZ, USA) and washed with 0.3% BSA in GBSS. After centrifugation, the cell pellet was resuspended in 0.3% BSA/GBSS and mixed with an equal volume of 28.7% Nycodenz (#AN1002423, Accurate Chemical, Carle Place, NY, USA) prepared in GBSS without NaCl. An amount of 0.3% BSA/GBSS was gently layered on top of the cell–Nycodenz suspension to create a discontinuous gradient. The gradient was centrifuged at 1400 *g* for 20 min at 4 °C with no brake. Cells were collected from the interface between the Nycodenz and GBSS layers and washed with 0.3% BSA/GBSS. Primary PSC lines between passages 2 and 4 were used for all experiments.

For 3D cultures, 4.4 × 10^4^ PSCs were embedded in Matrigel (#356230, Corning, Glendale, AZ, USA) in a transwell insert (#662610, Greiner Bio-One, Monroe, NC, USA), and 4.4 × 10^4^ mT3 pancreatic cancer cells were embedded in Matrigel in the lower compartment of a 24-well plate containing DMEM with 5% FBS and 1% penicillin–streptomycin. Conditioned media (CM) were collected after 72 h of culture and stored at −80 °C until use.

### 2.4. BMDM Generation and Culture

Bone marrow was isolated from the femurs of WT mice (#000664, Jackson Laboratory, Bar Harbor, ME, USA) as previously described [5]. The isolated bone marrow cells were cultured for 5 days in DMEM (#MT10017CV, Corning, Glendale, AZ, USA) supplemented with 10% FBS (#A5209501, Gibco, Waltham, MA, USA), 1% penicillin–streptomycin (#15140122, Gibco, Waltham, MA, USA), and 25 ng/mL MCSF (#3150250UG, Gibco, Waltham, MA, USA). The resulting BMDMs (8.0 × 10^4^ cells per well in 48-well plates) were treated with CM under 21% O_2_ or 1% O_2_ for 24 h and then harvested for RNA extraction. CM was added to BMDMs without dilution or concentration.

For IL-6 neutralization experiments, BMDMs were cultured with CM in the presence of 10 μg/mL α-IL-6 antibody (#BE0046, BioXCell, Lebanon, NH, USA) or isotype control antibody (#504512, BioLegend, San Diego, CA, USA). For JAK inhibition, BMDMs were cultured with CM in the presence of 3 μM ruxolitinib (#11609, Cayman Chemical, Ann Arbor, MI, USA) or DMSO vehicle control.

### 2.5. RT-qPCR

Total RNA was isolated from cells using the RNeasy mini kit (#74104, Qiagen, Hilden, Germany). cDNA was synthesized using a High-Capacity cDNA Reverse Transcription Kit (#4368814, Applied Biosystems, Waltham, MA, USA). PCR reactions were performed using SYBR Green PCR reagents (#A25742, Applied Biosystems, Waltham, MA, USA) mixed with cDNAs and primers in a QuantStudio Real-Time PCR system (Applied Biosystems, Waltham, MA, USA). Expression levels were normalized by *Rn18s*. The following primer sequences were used: *Arg1* forward 5′-CATTGGCTTGCGAGACGTAGAC-3′ and reverse 5′-GCTGAAGGTCTCTTCCATCACC-3′; *Fn1* forward 5′-CTCGCTTTGACTTCACCACCA-3′ and reverse 5′-TCTCCTTCCTCGCTCAGTTCGTACT-3′; *Il6* forward 5′-TACCACTTCACAAGTCGGAGGC-3′ and reverse 5′-CTGCAAGTGCATCATCGTTGTTC-3′; *Il6r* forward 5′-CCGACCTGTATGGTCAAAGG-3′ and reverse 5′-TGGATGACGCATTGGTACTG-3′; *Il6st* forward 5′-CCCATGGGCAGGAATATAGA-3′ and reverse 5′-CATAATCCAAGATTTTCCCATTG-3′; *Rn18s* forward 5′-GTAACCCGTTGAACCCCATT-3′ and reverse 5′-CCATCCAATCGGTAGTAGCG-3′; *Spp1* forward 5′-GCTTGGCTTATGGACTGAGGTC-3′ and reverse 5′-CCTTAGACTCACCGCTCTTCATG-3′.

### 2.6. ELISA

For ELISA analysis of conditioned media, 3D cultures of PSCs and pancreatic cancer cells were grown under 21% O_2_ or 1% O_2_ for 72 h, and the resulting conditioned media were collected. The conditioned media were assayed for IL-6 using the mouse IL-6 ELISA kit (#431304, BioLegend, San Diego, CA, USA) according to the manufacturer’s instructions.

### 2.7. Single-Cell RNA Sequencing Analysis

The human and murine single-cell RNA sequencing (scRNA-seq) data were previously published and obtained from the following publicly available sources: human PDAC, NIH dbGaP# PHS002071.v1.p1 [16]; murine spontaneous PDAC, NIH GEO# GSM6127792 [17], GSE129455 [18], and GSM3577885 [19]. Datasets generated from different sources or run dates were batch-corrected using Seurat’s IntegrateData workflow [20], and merged datasets from Donahue et al. [21] were used for downstream analyses. All analyses were performed in R (v4.5.0) using Seurat (v5.2.1).

**Figure 1:**
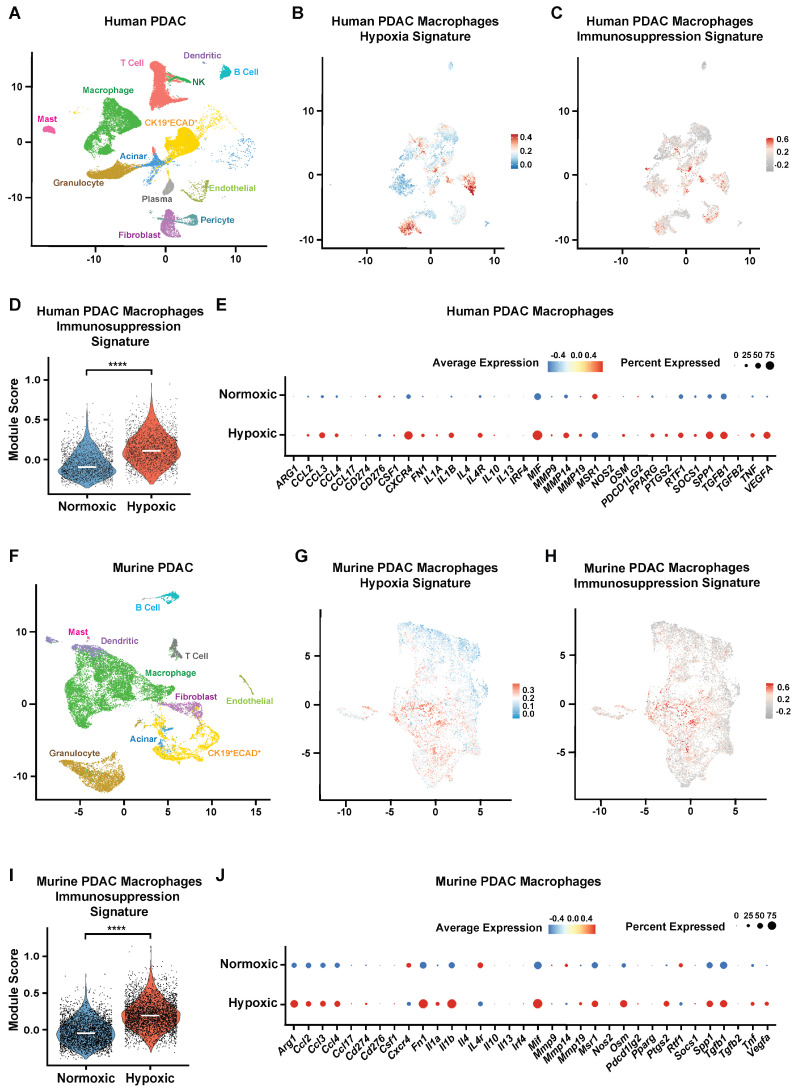
ScRNA-seq assessment of PDAC reveals hypoxic TAMs with elevated immunosuppression programs. (**A**–**E**) Human PDAC scRNA-seq analysis (*n* = 16 patients). (**A**) Uniform manifold approximation and projection (UMAP) showing major cell populations. The CK19^+^ (Cytokeratin19) ECAD^+^ (Ecadherin) cluster represents ductal/ductal-like malignant cells. (**B**) UMAP of macrophages colored by module score for the MSigDB Hallmark hypoxia signature (red, highest; blue, lowest). Macrophages were classified as hypoxic (top tertile) or normoxic (bottom tertile) based on the hypoxia module score. (**C**) UMAP of macrophages colored by immunosuppression gene signature score (red, highest; gray, lowest). (**D**) Violin plot showing immunosuppression signature scores in normoxic and hypoxic macrophages. (**E**) Dot plot showing relative expression of immunosuppression signature genes in normoxic and hypoxic macrophages. (**F**–**J**) Murine PDAC scRNA-seq analysis (*n* = 3 mice). (**F**) UMAP showing major cell populations. (**G**) UMAP of macrophages colored by Hallmark hypoxia module score (red, highest; blue, lowest). Hypoxic and normoxic macrophages were defined using the same tertile-based criteria applied to the human dataset. (**H**) UMAP of macrophages colored by immunosuppression gene signature score (red, highest; gray, lowest). (**I**) Violin plot showing immunosuppression signature scores in normoxic and hypoxic macrophages. (**J**) Dot plot showing relative expression of immunosuppression signature genes in normoxic and hypoxic macrophages. *p*-values were determined by the Mann–Whitney test with Bonferroni correction (**D**,**I**). **** *p* < 0.0001.

Hypoxia signature scores were calculated using the MSigDB “HALLMARK_HYPOXIA” gene set and Seurat’s AddModuleScore function. Fibroblasts and macrophages were independently stratified into tertiles based on hypoxia module scores; cells in the top tertile were classified as hypoxic and those in the bottom tertile as normoxic. Differential gene expression between hypoxic and normoxic populations was assessed using FindMarkers with the Mann–Whitney test and Bonferroni correction. Immunosuppression (34 genes, curated from the literature [7,22,23,24]; Figure 1E) and MSigDB HALLMARK_IL6_JAK_STAT3_SIGNALING signature scores were calculated using AddModuleScore. To avoid overlap with the HALLMARK_HYPOXIA gene set, *CXCR4*, *MIF*, and *VEGFA* were excluded from the Immunosuppression signature, and HMOX1, *IL6*, *JUN*, and *PIM1* were excluded from the HALLMARK_IL6_JAK_STAT3_SIGNALING signature prior to scoring. Differential signature enrichment between hypoxic and normoxic populations was determined using the Mann–Whitney test with Bonferroni correction.

### 2.8. Statistical Analysis

Data were analyzed using GraphPad Prism 10 software. Normality of data distribution was tested using the D’Agostino-Pearson test and/or the Shapiro–Wilk test. *T*-test, Mann–Whitney test, one-way ANOVA, and two-way ANOVA were performed as indicated in figure legends, based on data distribution and number of groups and variables tested. Holm–Sidak post hoc correction was applied for multiple comparisons. *p*-value < 0.05 was considered statistically significant. No statistical method was used to predetermine sample sizes, experiments were not randomized, and the investigators were not blinded to allocation during experiments and outcome assessment.

## 3. Results

### 3.1. Hypoxic TAMs Exhibit Elevated Immunosuppressive Molecular Programs in PDAC

We and others have observed substantial intratumoral heterogeneity of hypoxia in PDAC [12,25]. To characterize TAMs residing in hypoxic tumor regions in vivo, we analyzed a scRNA-seq dataset [16] comprising 16 human PDAC tumors (Figure 1A). Macrophages were stratified based on hypoxia exposure by mapping the Hallmark hypoxia gene set (MSigDB; 200 genes) [26] onto the scRNA-seq data (*n* = 16) [16] using gene module scoring (Figure 1B). TAMs were classified as hypoxic if their module score fell within the top tertile and normoxic if within the bottom tertile.

To assess the immunosuppressive potential of TAMs, normoxic and hypoxic TAM clusters were scored using a curated immunosuppressive gene signature [7,22,23,24] (Figure 1C). Of the 34 genes in this signature, *CXCR4*, *MIF*, and *VEGFA* were excluded to prevent confounding when calculating the immunosuppression signature score, as they are also components of the Hallmark hypoxia gene set. Our analysis revealed that hypoxic TAMs exhibit significantly higher immunosuppressive gene signature scores than normoxic TAMs (Figure 1D,E).

Consistent with these findings, analysis of a murine scRNA-seq dataset [17,18,19] of autochthonous PDAC tumors (*n* = 3) also showed elevated immunosuppressive molecular programs in hypoxic TAMs relative to normoxic TAMs (Figure 1F–J). Together, these data demonstrate that hypoxic TAMs are strongly associated with enhanced immunosuppressive programs in both human and mouse PDAC.

### 3.2. Fibroblast Reprogramming by Hypoxia Promotes Immunosuppressive Macrophage Phenotypes

ARG1 expression in TAMs drives immunosuppression in PDAC by depleting arginine and suppressing T cell activation [27]. Hypoxia can directly induce ARG1 expression in macrophages via hypoxia-inducible factors (HIFs) [28,29,30]. In addition to these intrinsic effects, hypoxia may also regulate macrophages indirectly through paracrine signals from neighboring cells [31,32]. Because hypoxia promotes an iCAF phenotype characterized by increased cytokine and chemokine production [12,33], we hypothesized that hypoxia-reprogrammed CAFs may enhance macrophage ARG1 expression.

**Figure 2:**
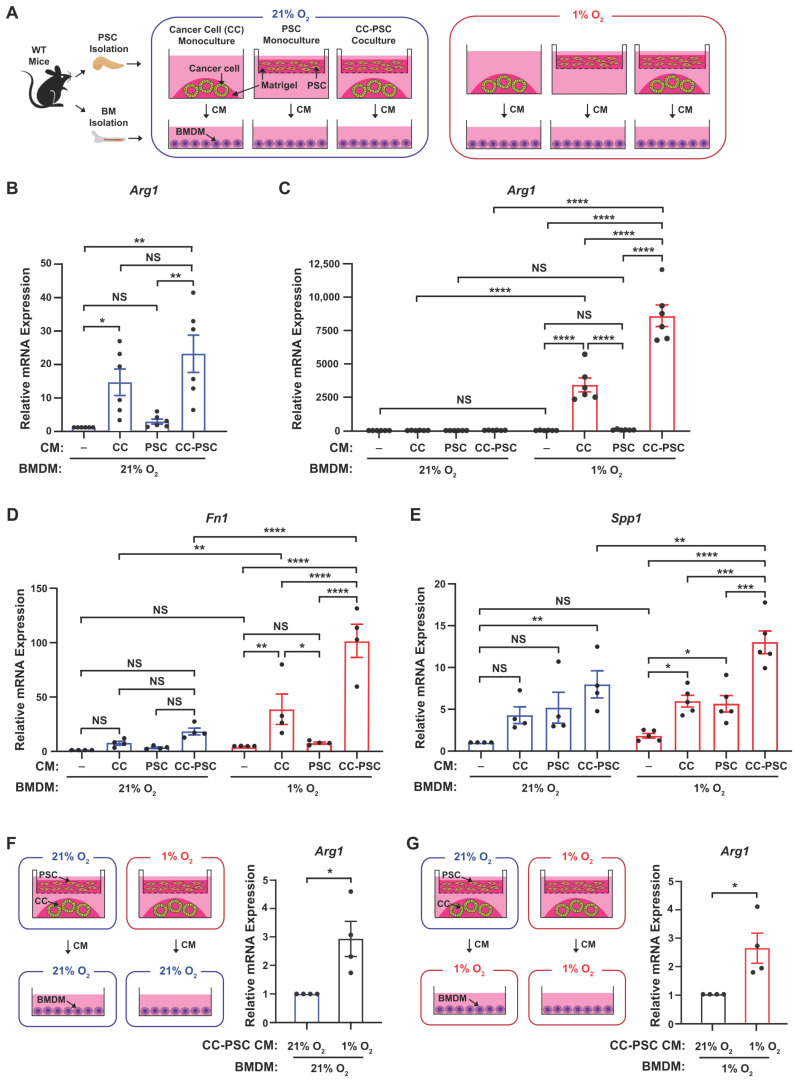
Hypoxia-induced fibroblast-derived factors promote expression of immunosuppressive macrophage markers. (**A**) Experimental scheme for BMDM treatment with conditioned media (CM). Pancreatic cancer cells (CC; mT3 line, derived from the *Kras^LSL-G12D/+^*; *Trp53^LSL-R172H/+^*; *Pdx1-Cre* mouse model of PDAC) and primary mouse PSCs were cultured independently or in coculture (CC-PSC) under normoxia (21% O_2_) or hypoxia (1% O_2_) for 72 h. CM collected from these cultures was transferred to BMDMs, which were then exposed to the indicated O_2_ levels for 24 h prior to RNA analysis. (**B**) RT-qPCR analysis of *Arg1* in BMDMs treated with normoxic CM from CC, PSC, or CC-PSC cultures and exposed to 21% O_2_ for 24 h (*n* = 6). Fresh media (-) served as the naive BMDM control. (**C**) RT-qPCR analysis of *Arg1* in BMDMs treated with hypoxic CM from CC, PSC, or CC-PSC cultures and exposed to 1% O_2_ for 24 h (*n* = 6). Normoxic BMDM data from (**B**) are included for comparison. (**D**,**E**) RT-qPCR analysis of *Fn1* (**D**) and *Spp1* (**E**) in BMDMs treated with CM from CC, PSC, or CC-PSC cultures and exposed to matched normoxic or hypoxic conditions for 24 h (*n* = 6). (**F**) Experimental scheme and RT-qPCR analysis of *Arg1* in BMDMs treated with normoxic or hypoxic CC-PSC CM and exposed to 21% O_2_ for 24 h (*n* = 4). (**G**) Experimental scheme and RT-qPCR analysis of *Arg1* in BMDMs treated with normoxic or hypoxic CC-PSC CM and exposed to 1% O_2_ for 24 h (*n* = 4). Data points represent independent biological replicates using distinct primary PSC lines and BMDMs isolated from different mice. Data are mean ± SEM. *p*-values were determined by one-way ANOVA with Holm–Sidak post hoc (**B**), two-way ANOVA with Holm–Sidak post hoc (**C**–**E**), and two-tailed unpaired *t*-test (**F**,**G**). NS, not significant. * *p* < 0.05; ** *p* < 0.01; *** *p* < 0.001; **** *p* < 0.0001.

To test this, we treated macrophages with conditioned media (CM) from 3D pancreatic cancer cell (CC)-PSC cocultures maintained under normoxia (21% O_2_) or hypoxia (1% O_2_). We used 1% O_2_ to model tumor hypoxia, reflecting the oxygen levels observed in human PDAC [13]. Primary PSCs isolated from wild-type mice were embedded in Matrigel within transwell inserts and cultured alone or with pancreatic tumor organoids in the lower chamber. Mono- and cocultures were exposed to normoxia or hypoxia, after which CM was collected and applied to bone marrow-derived macrophages (BMDMs). BMDMs were then cultured under matched O_2_ conditions—by pairing normoxic CM with normoxic BMDM culture and hypoxic CM with hypoxic BMDM culture—to model the respective tumor microenvironments (TME) (Figure 2A).

When BMDMs were cultured under normoxia, CM from CC monocultures and CC-PSC cocultures significantly increased *Arg1* expression compared with fresh media controls (Figure 2B). This induction was dramatically amplified when BMDMs were treated with the corresponding hypoxic CM and cultured under hypoxia (Figure 2C). In contrast, CM from PSC monocultures did not affect *Arg1* expression in either O_2_ condition (Figure 2B,C), consistent with our previous observation that the hypoxia-driven inflammatory fibroblast phenotype requires tumor cells. Notably, CM from hypoxic CC-PSC cocultures elicited significantly higher *Arg1* expression in BMDMs than CM from hypoxic CC monocultures (Figure 2C). In line with the *Arg1* results, BMDMs treated with hypoxic coculture CM and cultured under hypoxia exhibited significantly higher expression of additional immunosuppressive TAM markers, *Fn1* and *Spp1* [7,22,34,35], than BMDMs treated with normoxic coculture CM and cultured under normoxia (Figure 2D,E).

To further determine whether normoxic versus hypoxic CC-PSC cocultures differ in their capacity to regulate macrophage *Arg1*, we treated BMDMs with normoxic or hypoxic coculture CM under both O_2_ conditions. We observed that when BMDMs were cultured under normoxia, hypoxic coculture CM induced significantly higher *Arg1* expression than normoxic coculture CM (Figure 2F). Similarly, when BMDMs were cultured under hypoxia, hypoxic coculture CM elicited a significantly greater increase in *Arg1* levels than the normoxic coculture CM (Figure 2G). Collectively, these results suggest that hypoxia-mediated crosstalk between tumor cells and fibroblasts generates secreted factors—originating from or modulated by the fibroblast population—that promote immunosuppressive TAM marker expression. These factors may cooperate with the intrinsic hypoxic response of macrophages to drive an enhanced immunosuppressive phenotype.

### 3.3. IL-6 Is Primarily Expressed by CAFs in PDAC, and Hypoxia Increases Fibroblast IL-6 Expression

IL-6 is a key immunomodulatory cytokine implicated in immune cell polarization and has been shown to accelerate pancreatic tumor development and progression [36,37,38,39,40]. Given that hypoxia promotes IL-6 expression in CAFs through tumor cell-derived signals [12] and that CM from hypoxic CC-PSC cocultures increases expression of immunosuppressive macrophage markers (Figure 2C–G), we sought to determine the potential role of fibroblast-derived IL-6 in promoting immunosuppressive macrophage phenotypes within the hypoxic TME.

**Figure 3:**
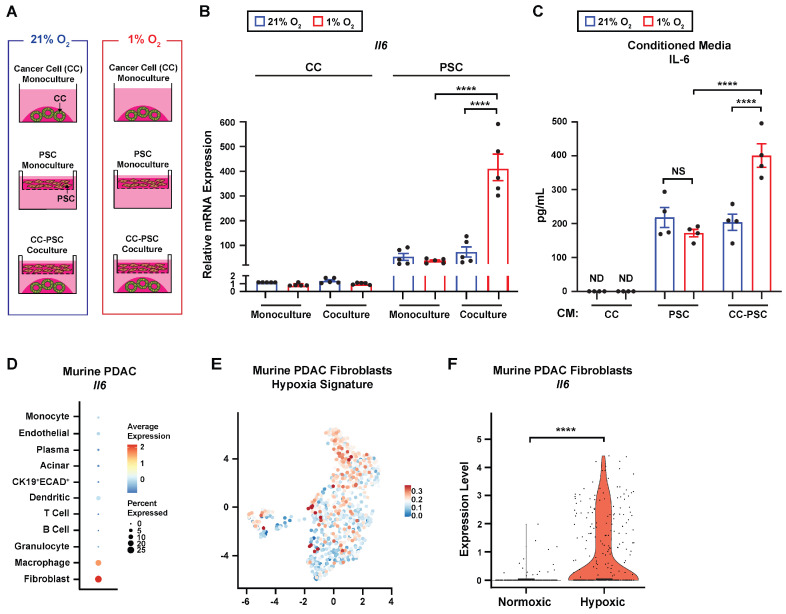
IL-6 is primarily expressed by CAFs and induced by hypoxia. (**A**) Schematic of the 3D monoculture and coculture platforms used to model CC-PSC interactions under normoxia and hypoxia. (**B**) RT-qPCR analysis of *Il6* in CCs and PSCs cultured alone (monocultures) or together (cocultures) under 21% O_2_ or 1% O_2_ for 48 h (*n* = 5). (**C**) ELISA of IL-6 in CM from monocultures and cocultures of CCs and PSCs under 21% O_2_ or 1% O_2_ for 72 h (*n* = 4). (**D**–**F**) Murine PDAC scRNA-seq analysis (*n* = 3 mice). (**D**) Dot plot showing *Il6* transcript levels across cell types. (**E**) UMAP of fibroblasts colored by module score for the Hallmark hypoxia signature (red, highest; blue, lowest). Fibroblasts were classified as hypoxic (top tertile) or normoxic (bottom tertile) based on module score. (**F**) Violin plot of *Il6* expression in normoxic and hypoxic fibroblasts. Data points in (**B**,**C**) represent independent biological replicates using individual primary PSC lines. Data in (**B**,**C**) are mean ± SEM. *p*-values were determined by two-way ANOVA with Holm–Sidak post hoc (**B**,**C**) and Mann–Whitney test with Bonferroni correction (**F**). ND, not detected; NS, not significant. **** *p* < 0.0001.

We first assessed IL-6 expression in pancreatic tumor organoids and PSCs under normoxic or hypoxic conditions (Figure 3A). qPCR analysis showed a significant increase in *Il6* mRNA levels in PSCs cocultured with CCs under hypoxia compared with normoxia (Figure 3B). Notably, hypoxia did not alter *Il6* expression in PSCs cultured alone, and *Il6* expression was substantially higher in PSCs than in CCs across all culture conditions (Figure 3B). Consistent with these observations, ELISA analysis revealed that IL-6 protein levels were significantly elevated in hypoxic CC-PSC coculture CM relative to normoxic coculture CM and hypoxic PSC monoculture CM (Figure 3C). Furthermore, IL-6 protein remained undetectable in CC monoculture CM regardless of O_2_ tension (Figure 3C). These data suggest that hypoxia promotes IL-6 expression in PSCs through tumor cell–PSC crosstalk.

To identify the source of IL-6 in PDAC in vivo, we evaluated *Il6* expression across different cellular compartments using the murine PDAC scRNA-seq dataset (*n* = 3) [17,18,19]. The results revealed the highest *Il6* expression in fibroblasts, followed by macrophages (Figure 3D). Fibroblasts also exhibited the largest proportion of *Il6*-expressing cells (Figure 3D). Of note, *IL6* transcripts were detected at very low frequency in the human PDAC scRNA-seq dataset, limiting reliable quantitative analysis.

Given that CAFs represent the predominant IL-6 source in PDAC and that hypoxia upregulates IL-6 in PSCs, we next examined the relationship between hypoxia and *Il6* expression in fibroblasts in PDAC in vivo. To do this, we assessed *Il6* levels in normoxic and hypoxic fibroblast clusters from the murine PDAC scRNA-seq dataset (*n* = 3) [17,18,19], stratified by the module score for the Hallmark hypoxia signature (MSigDB, 200 genes) [26] (Figure 3E). Hypoxic fibroblasts exhibited significantly higher *Il6* expression compared with normoxic fibroblasts (Figure 3F), indicating a positive correlation between hypoxia and *Il6* expression in fibroblasts within PDAC. Together, these data suggest that CAFs represent a major source of IL-6, and hypoxia promotes CAF IL-6 expression.

### 3.4. IL6/JAK/STAT3 Signaling Signature Is Enriched in Hypoxic TAMs in PDAC

**Figure 4:**
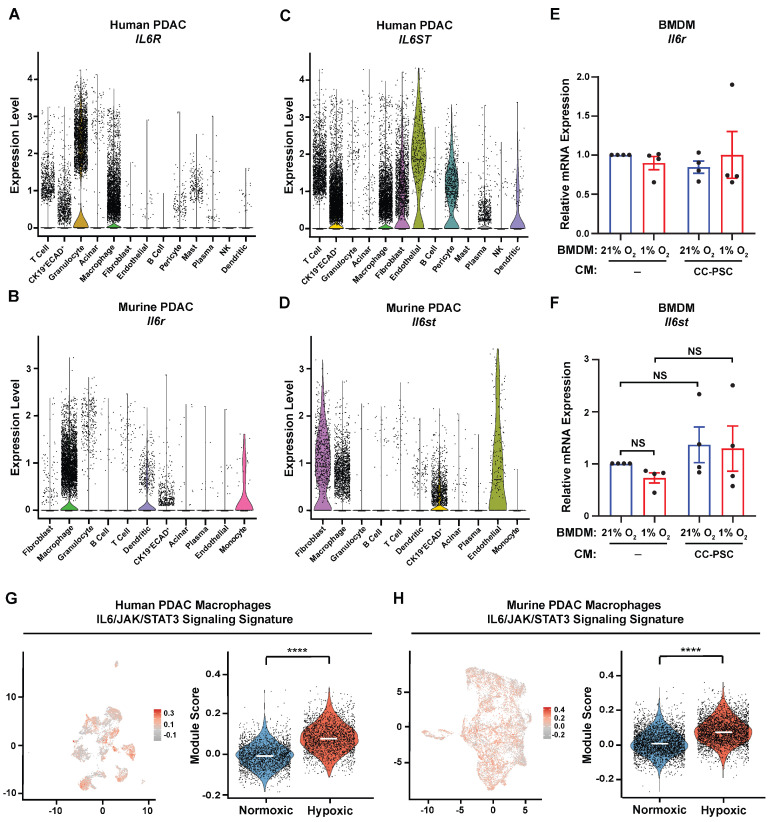
Hypoxic TAMs are enriched for the IL6/JAK/STAT3 signaling signature in PDAC. (**A**,**B**) Violin plots showing transcript levels of *IL6R* in the human PDAC scRNA-seq dataset (**A**) and *Il6r* in the murine PDAC scRNA-seq dataset (**B**). (**C**,**D**) Violin plots showing transcript levels of *IL6ST* in the human PDAC scRNA-seq dataset (**C**) and *Il6st* in the murine PDAC scRNA-seq dataset (**D**). (**E**,**F**) RT-qPCR analysis of *Il6r* (**E**) and *Il6st* (**F**) in BMDMs treated with fresh media (-) or CM from normoxic or hypoxic CC-PSC cocultures and exposed to matched O_2_ conditions for 24 h (*n* = 4). Data points represent independent biological replicates using distinct primary PSC lines and BMDMs isolated from different mice. Data are mean ± SEM. *p*-values were determined by two-way ANOVA. NS, not significant. (**G**,**H**) UMAPs of macrophages colored by IL6/JAK/STAT3 signaling signature score (red, highest; gray, lowest) and violin plots showing signature scores in normoxic and hypoxic macrophages in the human (**G**) and murine (**H**) PDAC scRNA-seq datasets. *p*-values were determined by the Mann–Whitney test with Bonferroni correction. **** *p* < 0.0001. NS, not significant.

IL-6 signals by binding to its receptor IL-6R, inducing homodimerization of gp130 (encoded by *IL6ST*), and subsequently activating the JAK/STAT signaling pathway [39]. To determine whether CAF-derived IL-6 can engage IL-6R/gp130 on TAMs in PDAC, we analyzed the expression patterns of *IL6R* and *IL6ST* in the human and murine PDAC scRNA-seq datasets. *IL6R* was most highly expressed in myeloid populations in both human and mouse PDAC (Figure 4A,B). Macrophages also expressed *IL6ST* in both datasets (Figure 4C,D), indicating that TAMs are competent to respond to IL-6. qPCR analysis of BMDMs showed that *Il6r* and *Il6st* are expressed, and their expression is not altered by hypoxia or CC-PSC coculture CM (Figure 4E,F).

Given that CAFs are a primary source of IL-6 in PDAC, that hypoxia increases CAF IL-6 production, and that TAMs express the receptor machinery required for IL-6 signaling, we hypothesized that the IL6/JAK/STAT axis is preferentially activated in TAMs residing within hypoxic tumor niches. To assess the in vivo relationship between hypoxic TAMs and JAK/STAT pathway activation, we scored cells from the normoxic and hypoxic TAM clusters using the MSigDB Hallmark IL6/JAK/STAT3 signaling signature [26]. Of the 87 genes in this signature, *HMOX1*, *IL6*, *JUN*, and *PIM1* were excluded to avoid confounding when calculating the IL6/JAK/STAT3 signaling signature score, as they are also components of the Hallmark hypoxia gene set. Our analysis revealed that the IL6/JAK/STAT3 signaling signature was significantly enriched in hypoxic TAMs compared with normoxic TAMs in both human and mouse PDAC (Figure 4G,H). Together, these findings indicate that hypoxic TAMs are associated with increased IL6/JAK/STAT3 pathway activity in PDAC.

### 3.5. Fibroblast-Derived IL-6 Mediates Hypoxia-Induced Immunosuppressive Macrophage Phenotype via JAK/STAT Signaling

**Figure 5:**
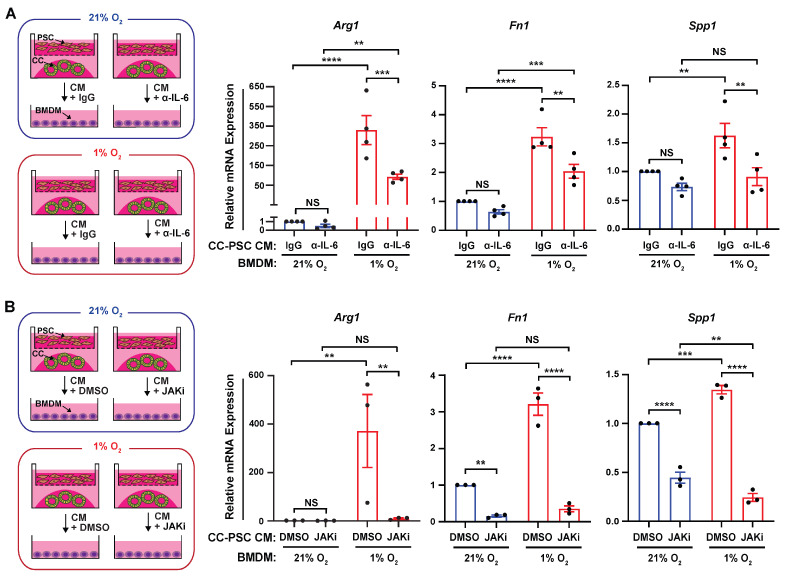
Fibroblast IL-6 promotes immunosuppressive macrophage phenotypes under hypoxia via JAK/STAT signaling. (**A**) Experimental scheme and RT-qPCR analysis of *Arg1*, *Fn1*, and *Spp1* in BMDMs treated with normoxic or hypoxic CC-PSC CM in the presence of an IL-6-neutralizing antibody (α-IL-6, 10 μg/mL) or an isotype control, and exposed to matched O_2_ conditions for 24 h (*n* = 4). (**B**) Experimental scheme and RT-qPCR analysis of *Arg1*, *Fn1*, and *Spp1* in BMDMs treated with normoxic or hypoxic CC-PSC CM in the presence of the JAK1/2 inhibitor ruxolitinib (JAKi, 3 μM) or DMSO vehicle control, and exposed to matched O_2_ conditions for 24 h (*n* = 3). Data points represent independent biological replicates using distinct primary PSC lines and BMDMs isolated from different mice. Data are mean ± SEM. *p*-values were determined by two-way ANOVA. NS, not significant. ** *p* < 0.01; *** *p* < 0.001; **** *p* < 0.0001.

To determine whether fibroblast-derived IL-6 is required for hypoxia-induced immunosuppressive macrophage polarization, we treated BMDMs with hypoxic CC-PSC coculture CM in the presence of an IL-6-neutralizing antibody or an isotype control, and exposed these BMDMs to hypoxia. Normoxic control counterparts were also included. As expected, in the presence of the isotype control, hypoxic BMDMs treated with hypoxic CM exhibited dramatically higher *Arg1* induction than normoxic BMDMs treated with normoxic CM (Figure 5A). However, under hypoxic conditions, neutralizing IL-6 in the hypoxic CM significantly attenuated *Arg1* induction in BMDMs compared with those treated with isotype control CM (Figure 5A). In contrast, IL-6 neutralization did not significantly alter *Arg1* expression in normoxic BMDMs treated with normoxic CM (Figure 5A). Consistent with these findings, IL-6 neutralization reduced expression of *Fn1* and *Spp1* in hypoxic BMDMs treated with hypoxic CM, but not in normoxic BMDMs treated with normoxic CM (Figure 5A). Of note, *Il6* expression in BMDMs was substantially lower than that in cancer cells and PSCs (Figure S1), suggesting that macrophage-derived IL-6 contributes minimally in this context.

To determine whether macrophage ARG1 induction by hypoxia depends on JAK/STAT signaling, we treated BMDMs with the JAK1/2 inhibitor ruxolitinib under matched O_2_ and CM conditions, with DMSO used as a vehicle control. JAK inhibition markedly reduced expression of *Arg1*, *Fn1*, and *Spp1* in BMDMs treated with the hypoxic CC-PSC coculture CM and exposed to hypoxia (Figure 5B). Collectively, these findings demonstrate that hypoxia-induced IL-6 production by fibroblasts promotes immunosuppressive macrophage phenotypes through activation of the JAK/STAT pathway.

## 4. Discussion

A defining feature of PDAC is its highly desmoplastic, immunosuppressive, and hypoxic TME [2,3]. Our analysis of human and murine scRNA-seq datasets revealed a robust association between hypoxia and immunosuppressive programs in TAMs within PDAC. Using 3D coculture systems of pancreatic cancer cells and PSCs, we demonstrate that hypoxia-driven fibroblast reprogramming promotes immunosuppressive macrophage phenotypes. These findings highlight a key role for CAFs in shaping TAM polarization and function in the hypoxic PDAC microenvironment.

Hypoxia regulates immune responses in a cell-type- and context-dependent manner and is largely immunosuppressive in cancer [28,41,42]. Within the hypoxic TME, TAM phenotype is regulated not only by cell-intrinsic hypoxic responses but also by extrinsic signals from surrounding cells, including tumor cells and CAFs, which are themselves responsive to hypoxia [12,43,44]. Thus, TAM phenotype reflects the integration of intrinsic hypoxic signaling and dynamically regulated paracrine inputs. By dissecting intrinsic regulation from extrinsic signals in vitro, we show that hypoxia upregulates fibroblast IL-6 expression through tumor cell–fibroblast interactions. This extrinsic signal cooperates with macrophage-intrinsic hypoxic responses to drive immunosuppressive phenotypes via activation of the JAK/STAT pathway.

Our scRNA-seq data analysis complements the coculture findings by showing that fibroblasts represent a major source of IL-6 in PDAC and that hypoxic fibroblast clusters exhibit increased *Il6* expression. In parallel, hypoxic TAMs display enrichment of IL6/JAK/STAT3 signaling signatures in PDAC. While scRNA-seq is a powerful tool for studying the TME, it remains subject to technical biases; therefore, future studies using multiplex immunohistochemistry on human PDAC samples will be necessary to validate these findings at the protein level and within the spatial architecture of the tissue. Furthermore, recent studies of macrophage-to-CAF signaling [44,45], together with our findings, underscore the importance of defining hypoxia-regulated bidirectional CAF–macrophage crosstalk.

Interestingly, although IL-6 neutralization in conditioned media from hypoxic tumor cell–PSC cocultures significantly attenuated the expression of immunosuppressive TAM markers, it did not fully restore them to normoxic levels. In contrast, pharmacological inhibition of JAK signaling abrogated hypoxia-induced expression of these markers. These findings suggest that multiple hypoxia-regulated fibroblast-derived factors may contribute to macrophage polarization by converging on the JAK/STAT pathway, and that this axis may also integrate macrophage-intrinsic responses to hypoxia. Future studies will aim to identify additional tumor cell- or fibroblast-derived factors that cooperate with IL-6 to regulate macrophage function within the hypoxic TME. One potential candidate is CXCL1, which has been reported to promote M2 macrophage polarization in PDAC [46], is induced by hypoxia in pancreatic fibroblasts [12], and can activate the JAK/STAT pathway [47].

In summary, our study demonstrates a strong association between hypoxia and immunosuppressive TAM phenotypes in PDAC and supports a model in which hypoxia promotes their immunosuppressive phenotype through fibroblast-derived IL-6 and subsequent activation of JAK/STAT signaling. Our findings reveal a paracrine mechanism by which hypoxia coordinates tumor cell–fibroblast–macrophage interactions to drive immune evasion in PDAC.

## Data Availability

All data needed to evaluate the conclusions in the paper are present in the paper and/or the Supplementary Materials. This study did not generate new materials.

## Supplementary Materials

The following supporting information can be downloaded at: https://www.mdpi.com/article/10.3390/cells15080683/s1, Figure S1: BMDMs exhibit minimal *Il6* expression relative to pancreatic cancer cells and PSCs.
