# Supplementary material for: Hypoxia-Induced Fibroblast IL-6 Promotes Immunosuppressive Macrophage Phenotypes in Pancreatic Cancer
Source: Cells. 2026 Apr 13;15(8):683. doi: 10.3390/cells15080683 (PMC13114822; doi:10.3390/cells15080683)
Supplement: Supplementary file 1 [file cells-15-00683-s001.zip › cells-4232606-supplementary.pdf]

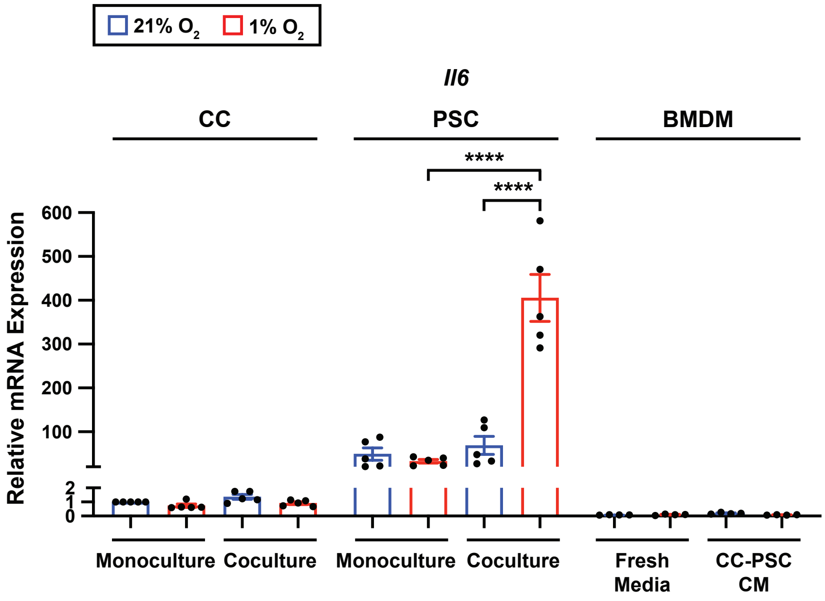

Figure S1. BMDMs exhibit minimal *Il6* expression relative to pancreatic cancer cells and PSCs. RT-qPCR analysis of *Il6* in BMDMs treated with fresh media or CM from normoxic or hypoxic CC-PSC cocultures and exposed to matched O_2 conditions for 24 hours (n = 4). CC and PSC data from Figure 3B are included for comparison. Data points represent independent biological replicates using distinct primary PSC lines and BMDMs isolated from different mice. Data are mean ± SEM. *P* values were determined by two-way ANOVA. $^{****}p < 0.0001$.
